# Optic Nerve Head Development in Healthy Infants and Children Using Handheld Spectral-Domain Optical Coherence Tomography

**DOI:** 10.1016/j.ophtha.2016.06.057

**Published:** 2016-10

**Authors:** Aarti Patel, Ravi Purohit, Helena Lee, Viral Sheth, Gail Maconachie, Eleni Papageorgiou, Rebecca J. McLean, Irene Gottlob, Frank A. Proudlock

**Affiliations:** 1 Ulverscroft Eye Unit, University of Leicester, Leicester, United Kingdom; 2 Clinical and Experimental Sciences, Faculty of Medicine, University of Southampton, Sir Henry Wellcome Laboratories, Southampton, United Kingdom; 3 Department of Ophthalmology, University Hospital of Larissa, Mezourlo, Larissa, Greece

**Keywords:** BMO-MRW, Bruch's membrane opening–minimum rim width, C/D_fund_, cup-to-disc ratio described by fundoscopy, C/D_OCT_, cup-to-disc ratio defined using OCT measurements, D, diopter, ICC, intraclass correlation coefficient, ILM, inner limiting membrane, OCT, optical coherence tomography, ONH, optic nerve head, RNFL, retinal nerve fiber layer, SD, spectral-domain

## Abstract

**Purpose:**

To determine feasibility of optic nerve head (ONH) imaging and to characterize ONH development in full-term infants without sedation using handheld spectral-domain optical coherence tomography (SD OCT).

**Design:**

Prospective cross-sectional study.

**Participants:**

Three hundred fifty-two children aged between 1 day and 13 years.

**Methods:**

All participants were imaged using handheld SD OCT without sedation during a single scan session. The percentage of successful scans was calculated. Interexaminer reproducibility and differences between right and left eyes were assessed using intraclass correlation coefficients (ICCs). Images were analyzed using ImageJ software. The developmental trajectories over time for ONH parameters were calculated using fractional polynomial modelling.

**Main Outcome Measures:**

Disc and cup diameter (expressed as distance in micrometers and visual angle in degrees), cup depth, Bruch's membrane opening–minimum rim width (BMO-MRW), retinal thickness, and retinal nerve fiber layer (RNFL; 1700 μm and 6° from the disc center).

**Results:**

On average, 70% of participants were imaged successfully. Interexaminer reliability was excellent (ICC, >0.89) for diametric and retinal thickness parameters. Right and left eyes were similar for diametric measurements (ICC, >0.79), but more variable for nasal BMO-MRW, RNFL, and retinal thickness. The mean disc and cup diameter increase by 30% and 40%, respectively, between birth and 13 years of age when expressed as a distance measure, but remained constant (at 5°–5.5° and 2°, respectively) when expressed as a visual angle with reference to the eye nodal point. The peripapillary temporal RNFL demonstrated a marked initial decrease of nearly 35% between birth and approximately 18 months of age. This was followed by a slow increase up to 12 years of age when measured at 1700 μm from the disc center, although there was little change when measured at 6° from the disc center.

**Conclusions:**

We demonstrated feasibility of handheld SD OCT imaging of the ONH in full-term infants and children without anaesthesia or sedation. This is the first in vivo handheld SD OCT study to describe the development of ONH parameters during the critical early years of visual maturation. Our results provide a normative database for use in routine practice and further studies of ONH pathologic features.

The appearance of the optic nerve head (ONH) can indicate both ocular and central nervous tissue pathologic features. Although the ONH retains some plasticity during the early years of life, damage to the ONH can result from conditions such as pediatric glaucoma, one of the leading causes of childhood blindness worldwide.^1^, ^2^ Other conditions, including central nervous system tumors, craniofacial syndromes, neuropathies, and congenital anomalies, also can involve the visual pathway including the optic nerve and can affect vision.^3^, ^4^, ^5^

Clinical evaluation of the ONH is a combination of functional assessment (for example, visual field and color vision testing) and anatomic assessment (for example, direct visualization and imaging). In young children, many of these tests are not possible because of limited cooperation, communication, and fixation. Routine examination of the ONH often is restricted to funduscopy, providing a quick, noninvasive subjective assessment of ONH appearance. Until recently, fundus photography was the only method for quantifying the ONH of children objectively.

Optical coherence tomography (OCT), first described in 1991, has revolutionized imaging and the subsequent clinical management of many ocular conditions in adults.^6^ Current OCT machines with a theoretical axial resolution of a few micrometers can take a so-called optical biopsy of retinal structures within seconds in vivo and without direct ocular contact.

Optical coherence tomography studies of older children who are able to position at a head chin rest and maintain fixation have provided important information about ONH parameters in childhood. For example, the average retinal nerve fiber layer (RNFL) thickness measured with spectral-domain (SD) OCT in children between 5 and 18 years of age has been reported to be 102 to 113 μm, with only a small degree of change over this period.^7^, ^8^, ^9^, ^10^, ^11^ Critically, these studies miss the early years, during which time cadaveric and histologic studies suggest that most maturation takes place.^12^, ^13^

The recent advent of handheld ultra–high-resolution SD OCT has the potential to improve our understanding of ocular development and pediatric ophthalmic care, just as table-mounted OCT has accomplished for adult patients. Avery et al^14^ demonstrated excellent intravisit and intervisit reliability of optic nerve RNFL analysis for 59 children younger than 13 years imaged with handheld SD OCT. All participants were sedated during imaging and had a diagnosis of optic nerve glioma, neurofibromatosis type 1, or both. Studies of premature infants and full-term neonates imaged with handheld SD OCT without sedation have reported increased cup diameter associated with prematurity and thinning of the RNFL at increased arc distance from disc center, consolidating earlier studies using fundus photography.^15^, ^16^

The purpose of the current study was to create a clinically useful pathway for using the handheld SD OCT to assess ONH morphologic features of children in vivo. To achieve this, our aims were (1) to test the feasibility of handheld SD OCT for imaging the ONH in full-term children without sedation, (2) to estimate interexaminer reliability and intereye similarity of ONH measures in children, (3) to describe ONH development between birth and 13 years of age, and (4) to provide normative estimates with 95% confidence intervals for 13 age bands between birth and 13 years of age for ONH parameters that can be measured in a clinical setting for future studies of ocular development in children.

## Methods

### Participants

Three hundred fifty-two children up to 13 years of age born after 37 weeks' gestation and without prior neurologic or ophthalmic diagnoses were recruited to the study. Potential participants were all identified from the Leicestershire region within the National Health Service representing a varied socioeconomic background. All participants underwent ophthalmic examination including fundus examination and orthoptic assessment on the same day. Retinoscopy was performed after dilation with cyclopentolate 0.5% eye drops for younger participants. For older children who did not prefer to undergo dilation, uncyclopleged refraction was performed. Neonatal infants were examined in the delivery suite between 1 and 7 days after birth. Older babies and children were assessed during routine pediatric outpatient clinic appointments.

Participants were excluded if initial assessment revealed an ophthalmic abnormality or a spherical equivalent refractive error of more than ±3 diopters (D). Age-appropriate visual acuity assessments, including Keeler Acuity Cards (Windsor, Berkshire, UK) for preverbal children, Crowded LogMAR Kay Picture Tests (Tring, Hertfordshire, UK) for toddlers, and Snellen letters for older children, were performed before dilation. Participants unable to perform visual acuity assessment at recruitment were invited for repeat testing at a later date. Ten participants were excluded if initial or follow-up assessment demonstrated a visual acuity recording of less than the 99% confidence level for age.^17^, ^18^ Fifteen further participants did not return for repeat assessment and also were excluded. Five participants were excluded because of high refractive error, and 1 participant was excluded after review of medical notes found that the gestational age at birth was 36 weeks. Interexaminer reproducibility and intereye similarity were assessed for 30 participants in different age groups.

The study adhered to the tenets of the Declaration of Helsinki, and ethics committee approval was granted. Informed consent was obtained from all parents or guardians of participants, and older children also gave their assent to the study.

### Handheld Spectral-Domain Optical Coherence Tomography Image Acquisition

A noncontact handheld SD OCT device (ENVISU C class 2300; Bioptigen, Inc., Research Triangle Park, NC; axial resolution, 3.3 μm; scan depth, 3.4 mm; 32 000 A-scans per second) was used to image the ONH of all participants without sedation. Pacifiers and visual fixation devices, including cartoons played on screens, toys, and books, were used to minimize movement of the child during imaging. The acquisition protocol used a 10×5-mm scanning window. If the ONH was not imaged successfully, a larger 10×10-mm scanning window was used. The 3-dimensional raster scan program for both scan sequences consisted of 100 B-scans and 500 A-scans per B-scan line. The short acquisition time (2.9 seconds) enabled operators to obtain images of the ONH with minimal disruption of image quality.

### Image Analysis

**Figure 1:**
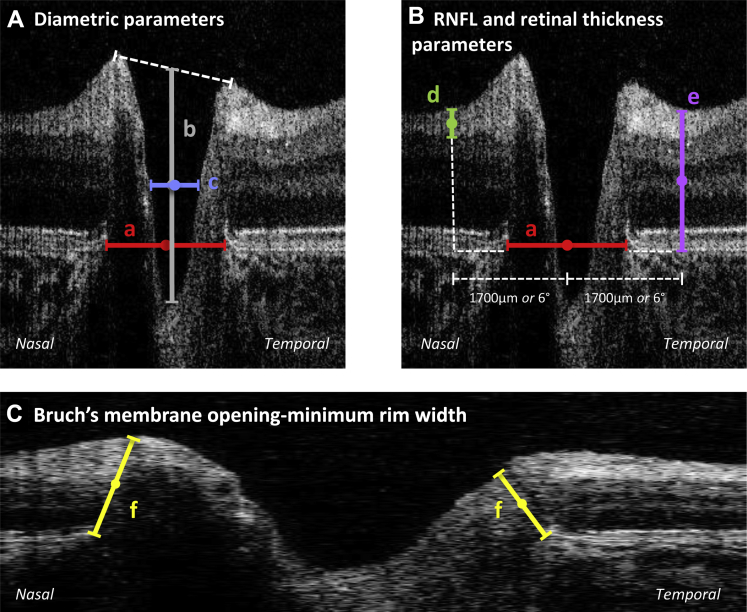
Optic nerve head tomograms of the left retina from an infant 9 months of age. **A**, Diametric parameters: (a) disc diameter, from nasal to temporal Bruch's membrane (red); (b) cup depth, vertical distance from cup base to midpoint of neuroretinal peaks (grey); (c) cup diameter, measured at midpoint of cup depth (blue). **B**, Peripapillary parameters measured at 1700 μm and 6.0° from central disc diameter: (d) retinal nerve fiber layer (RNFL), thickness measurement of hyperreflective tissue inferior to inner limiting membrane (ILM; green); (e) retinal thickness, thickness measurement from ILM to Bruch's membrane (purple). **C**, Natural scale image with basement membrane opening minimum rim width measurement: (f) yellow.

Analysis was based primarily on a single B-scan through the deepest part of the optic cup to derive normative estimates for parameters that can be measured mainly in a clinical setting without the need for custom software (i.e., can be measured mainly using calipers). The B-scan image was randomized before analysis and was analyzed by 1 of 2 assessors (A.P. and R.P.) masked to the age of the participant to minimize bias. B-scan images were assessed and analyzed (Fig 1) using ImageJ software version 1.48 (available at: http://imagej.nih.gov/ij/ and provided in the public domain by the National Institutes of Health, Bethesda, MD).

The edges of the Bruch's membrane were identified manually by the examiner and were used to define the optic disc diameter. A semiautomated ImageJ software program flattened the image and identified the contour of the inner limiting membrane (ILM) using the ABSnake plugin, which was corrected manually where necessary. The neuroretinal rim was measured using Bruch's membrane opening–minimum rim width (BMO-MRW), defined as the shortest distance between the disc edge (termination of Bruch's membrane) and the ILM. The optic cup diameter was calculated at half-cup depth from the axial distance between the height of the nasal and temporal neuroretinal rims to the optic cup base (Fig 1A). Comparison measurements for optic cup diameter were obtained at a plane 200 μm anterior to the disc. The cup-to-disc ratio calculated using OCT defined measurements has been termed C/DOCT to distinguish from that derived from fundoscopy in previous studies (C/DFUND). The total retina and RNFL thicknesses were calculated at 1.7 mm distance laterally after adjusting for axial length, which was estimated from the age of the participant using previously published literature.^19^ Lateral measurements (disc and cup width) also were calculated as visual angles using the formula: visual angle = tan^−1^ (retinal distance in millimeters/17 mm), where 17 mm represents the distance from nodal point to the retina in the adult eye.^20^ Total retina and RNFL thicknesses were measured at a 6° angle from the center of the optic disc. This was comparable with a retinal distance of approximately 1.26 mm at birth and 1.74 mm in adults.

### Feasibility Analysis

Successful images were defined as those that allow the visualization of the deepest section of the optic cup, retinal pigment epithelium edges, and contour of the ILM nasal and temporal to the optic disc.

### Analysis of Interexaminer Reliability and Intereye Similarity

Interexaminer reliability was analyzed by comparing assessments of the same ONH image by assessors 1 (A.P.) and 2 (R.P.). Intereye similarity also was assessed by analyzing ONH images of right and left eyes from the same participant imaged during a single visit. The same assessor (A.P.) analyzed both images. Interexaminer reproducibility and intereye similarity were described with intraclass correlation coefficients (ICCs) using SPSS software version 22.0 (SPSS, Inc., Chicago, IL).

### Statistical Modelling of Normal Optic Nerve Head Development

Fractional polynomial modelling was used to estimate: (1) the mean trajectory (±95% confidence interval) of development for each ONH parameter over time, and (2) the prediction interval, that is, the 95% confidence interval forecasted for a newly acquired measure for each ONH parameter over time. Statistical analysis was performed using STATA software version 13 (StataCorp LP, College Station, TX; available at: http://www.stata.com). Linear changes with log age were explored using linear mixed models to include eye recorded and repeated-measures recordings at different time points from the same participants in the model. Optic cup depth measures were log transformed to approximate more closely to a normal distribution.

### Normative Estimates

The fractional polynomials estimates were used to generate 95% prediction intervals for 13 age groups for ONH parameters. These values are expressed in both corrected formats (i.e., lateral measures rescaled using axial length appropriate for age) and uncorrected formats (i.e., using values given by the machine assuming scaling for a normal eye with axial length of 24 mm). The uncorrected values can be used as a reference with the existing software and caliper function available on the handheld SD OCT.

## Results

### Feasibility

The ONH of 1 eye was imaged successfully for 249 of the 352 infants and children. The average success rate was 70%, ranging between 53% and 100% across the different age groups, with the highest success rates achieved at older ages (Table 1, available at www.aaojournal.org). A scanning window of 10×5 mm and 10×10 mm successfully captured the ONH for 204 and 45 participants, respectively. Six images were excluded after detailed review of B-scan images with ImageJ software revealed the edges of the Bruch's membrane or optic cup base could not be identified. A further 25 participants were excluded because of reduced or absent visual acuity recordings. The remaining 218 participants included 122 female infants (50.2%) with a mean age of 3 years and 6 months (standard deviation, 3 years; range, 0–13 years). The sample included 70.4%, 19.4%, and 10.2% of participants within ±1.00 D, ±1.00 to 2.00 D, and ±2.00 to 3.00 D spherical equivalent, respectively.

### Analysis of Interexaminer Reliability and Intereye Similarity

Interexaminer reliability was high (>0.89) for diametric, retinal thickness and BMO-MRW parameters (Table 2). Nasal RNFL interexaminer reliability was found to be consistently higher than temporal RNFL for younger and older participants.

Right and left eyes also demonstrated similar values for disc and cup diameters (ICC, >0.793) with comparable cup-to-disc ratios (C/D_OCT_) between both eyes (ICC, 0.741). Retinal thickness and RNFL parameters were similar temporally (ICC, >0.713), but were reduced for nasal retinal thickness (ICC, 0.655; Table 2).

### Change in Optic Nerve Head Parameters with Age

**Figure 2:**
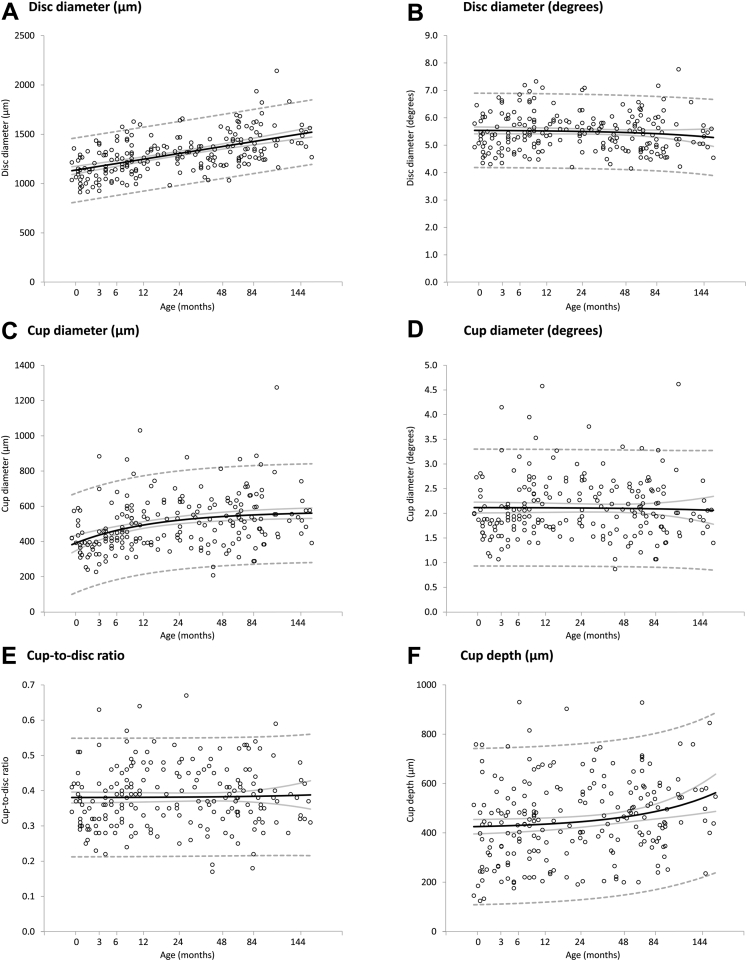
Scatterplots demonstrating the change of (**A**) optic disc diameter (in micrometers), (**B**) optic disc diameter (in degrees), (**C**) optic cup diameter (in micrometers), (**D**) optic cup diameter (in degrees), (**E**) cup-to-disc ratio, and (**F**) cup depth (in micrometers) with age (in months). Mean values *(black line*), 95% confidence intervals of the mean (*grey line*), and prediction interval (*dashed line*) are highlighted.

Optic disc diameter increased throughout childhood, demonstrating an approximately linear relationship between disc diameter and log age (linear mixed model: *F* = 105.29; *P* < 0.0001), indicating that the greatest progression is seen earlier in life (Fig 2A; Table 3). When expressed in micrometers, mean disc diameter increased by 30% between birth and 13 years of age, from a mean of 1142 μm to 1486 μm, respectively. In contrast, when expressed as a visual angle, there was no significant change in disc diameter with age (*F* = 0.01; *P* = 0.94), with the mean disc diameter remaining between 5° and 5.5° between birth and 13 years of age (Fig 2B).

Mean cup diameter measured at half cup depth (Fig 1A) also increased with age throughout childhood (increasing by 40% from a mean of 398 μm at birth to 557 μm at 13 years of age), with the greatest progression seen in the first 2 years of life (*F* = 29.28; *P* < 0.0001; Fig 2C; Table 3). Similar to disc diameter, cup diameter, when expressed as a visual angle, did not change significantly with age, but remained at approximately 2° (*F* = 0.72; *P* = 0.40; Fig 2D). The mean C/D_OCT_ remained stable between 0.381 and 0.386 (*F* = 0.72; *P* = 0.40; Fig 2E; Table 3).

**Figure 3:**
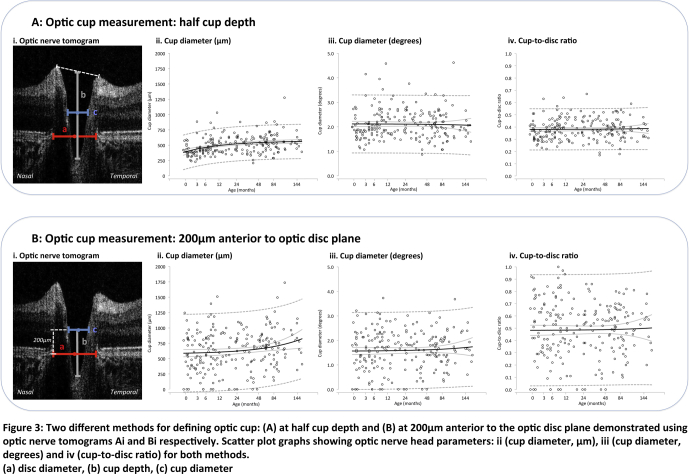


When defined 200 μm anterior than the disc plane, the cup diameter did not demonstrate a dramatic increase in the first few years of life; however, a small number of participants did show cupless discs (n = 11; Fig 3Bii, available at www.aaojournal.org). Neither method for defining cup diameter showed significant change when cup diameter was expressed as a visual angle or for C/D_OCT_, although the standard deviations were larger when using the 200-μm technique (Fig 3Aiii, Aiv, Biii, Aiv, available at www.aaojournal.org).

Mean cup depth increased by 22% between birth and 13 years of age (mean, from 426 μm to 532 μm, respectively), with a wide distribution of values across all ages (Fig 2F).

**Figure 4:**
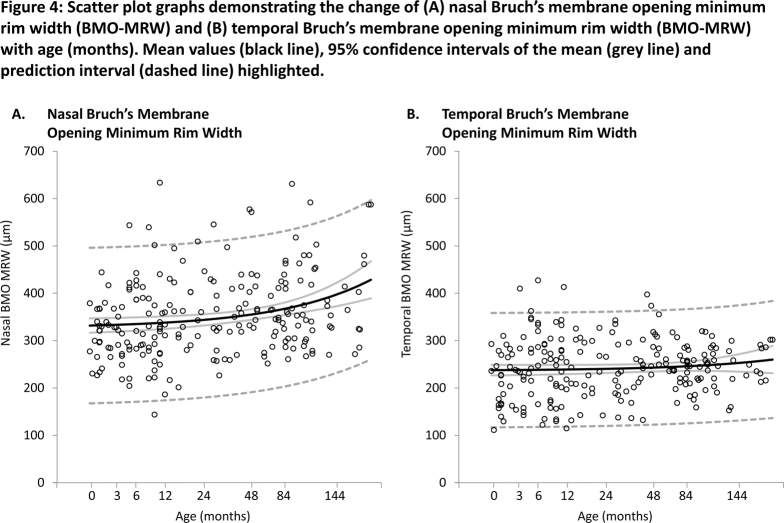


The nasal BMO-MRW showed a gradual increase with age (Fig 4A, available at www.aaojournal.org; overall change in linear mixed model: *F* = 17.33; *P* < 0.0001). The temporal BMO-MRW was considerably smaller than the nasal aspect and did not change significantly over time (Fig 4B, available at www.aaojournal.org; *F* = 2.26; *P* = 0.13).

### Change in Peripapillary Parameters with Age

**Figure 5:**
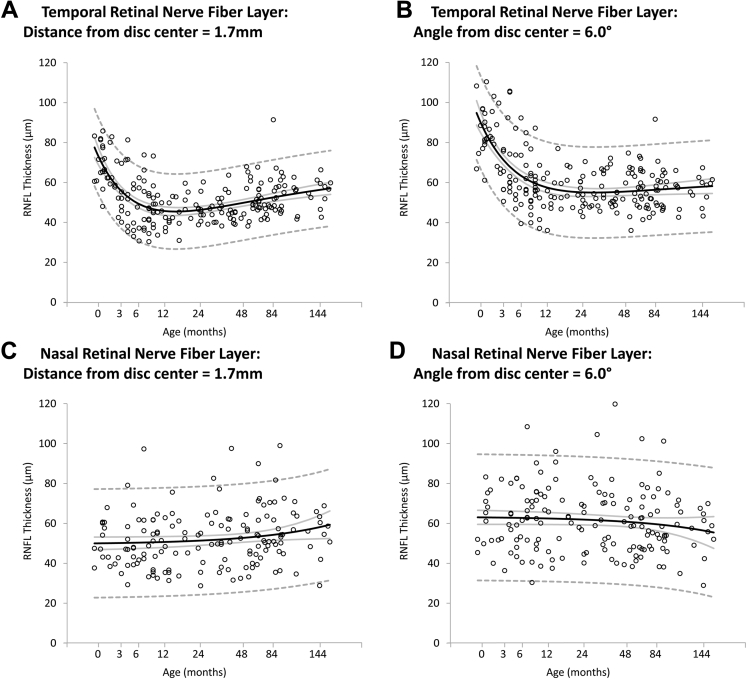
Scatterplots demonstrating the change of (**A**) temporal retinal nerve fiber layer (RNFL) measured at 1.7 mm from the disc center, (**B**) temporal RNFL measured at 6.0° from the disc center, (**C**) nasal RNFL measured at 1.7 mm from the disc center, and (**D**) nasal RNFL measured at 6.0° from the disc center with age (in months). Mean values (*black line*), 95% confidence intervals of the mean (*grey line*), and prediction interval (*dashed line*) are highlighted.

In contrast to the temporal rim, the temporal RNFL demonstrated a marked initial decrease of approximately 33% between birth and approximately 18 months of age (from a mean of 70 μm to 46 μm). When measured at a fixed distance of 1.7 mm from the disc center, this was followed by a slow increase up to 13 years of age (an increase of 22% up to 55 μm; Fig 5A; Table 4). However, when measuring temporal RNFL thickness at a fixed visual angle of 6° from the disc center (Fig 5B), after the initial decrease, the RNFL thickness remained constant at approximately 55 μm. The nasal RNFL did not change significantly when measured at a fixed distance of 1.7 mm (*F* = 2.77; *P* = 0.10; Fig 5C; Table 4) or a fixed angle of 6° (*F* = 1.95; *P* = 0.17; Fig 5D).

**Figure 6:**
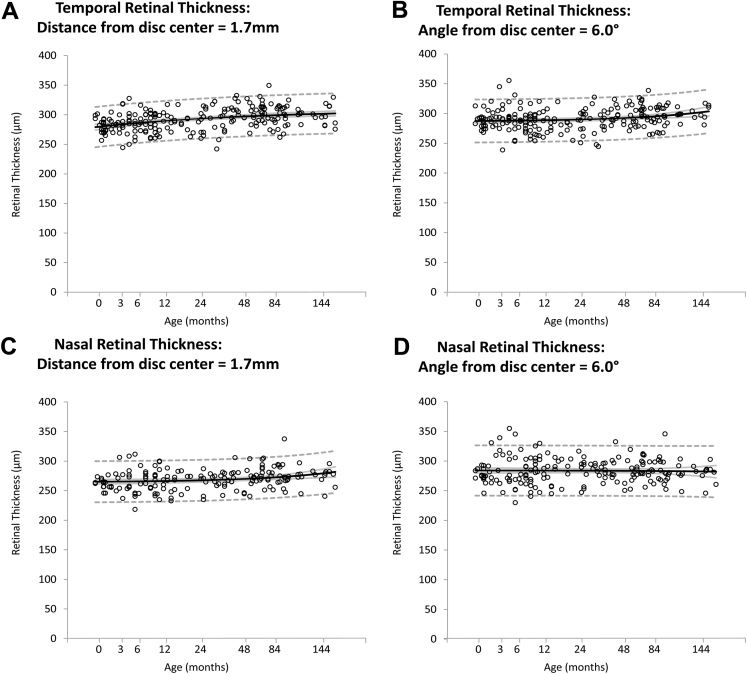
Scatterplots demonstrating the change of (**A**) temporal retinal thickness (RT) measured at 1.7 mm from the disc center, (**B**) temporal RT measured at 6.0° from the disc center, (**C**) nasal RT measured at 1.7 mm from the disc center, and (**D**) nasal RT measured at 6.0° from the disc center with age (in months). Mean values (*black line*), 95% confidence intervals of the mean (*grey line*), and prediction interval (*dashed line*) are highlighted.

When measured at a fixed distance of 1.7 mm from the disc center, retinal thickness demonstrated a small but highly significant increase between birth and 13 years of age both temporally (*F* = 31.8; *P* < 0.0001; Fig 6A) and nasally (*F* = 9.93; *P* = 0.002; Fig 6C; mean change between birth and 13 years of age, 7.4% and 4.8%, respectively). When expressed as a fixed visual angle of 6°, only temporal retinal thickness showed a significant difference in thickness throughout early infancy and childhood (*F* = 14.2; *P* < 0.001; Fig 6D), but there were no overall significant changes nasally (*F* = 0.02; *P* = 0.88; Fig 6B). Spherical equivalent was not a significant factor (*P* > 0.1) when included in statistical models for ONH or peripapillary parameters and did not affect the outcome of these models.

A schematic summary of the ONH and peripapillary development using handheld SD OCT can be seen in supplemental video 1 (available at www.aaojournal.org). The video illustrates dynamic changes in 2-dimensional space and shows that as disc diameter increases throughout childhood, the RNFL is shifted away from the ONH center. This leads to the temporal RNFL thickening observed later in development (e.g., 1700 μm temporally from the optic disc center). Consequently, the temporal RNFL thinning observed in early development is likely to be through an alternative process (see Discussion).

### Uncorrected Normative Estimates for Use with Caliper Function

**Figure 7:**
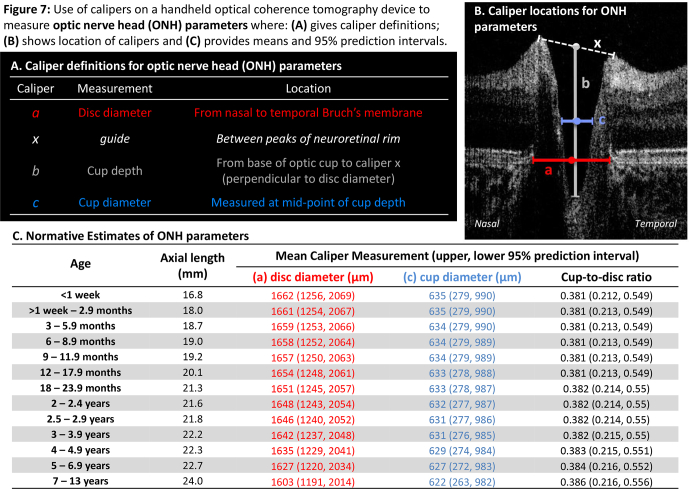


**Figure 8:**
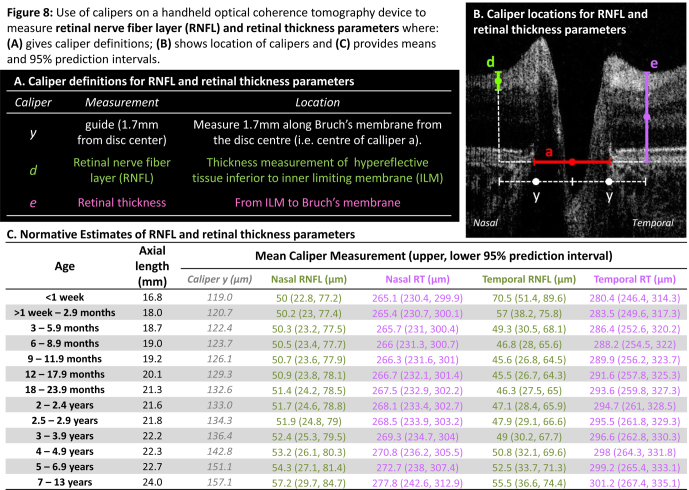


Optic nerve head parameters described in Tables 3 and 4 are corrected values rescaled using axial length appropriate for age. Calipers available on the handheld SD OCT device can be used to make quantitative assessments in children at the time of imaging. Supplemental Figures 7 and 8 (available at www.aaojournal.org) show where to position the calipers with an ONH tomogram. The reference values provided in Figures 7 and 8 are uncorrected for axial length for direct comparison to readings from the calipers (which assume an adult axial length of 24 mm).

## Discussion

The purpose of this in vivo handheld SD OCT study was to chart ONH development in healthy young children from birth to 13 years of age. All images were obtained from children without sedation in an outpatient setting to reflect clinical application of the system. Despite movement of the child, scan tilt, and different operators, the average success rate was 66% for children imaged between the neonatal stage and 5 years of age. In the same age group, we demonstrated excellent interexaminer reproducibility (ICC, 0.925–0.947 for diametric parameters), comparable with that of table-mounted OCT studies^11^ of older children (ICC between 0.858 and 0.972 for thickness and area parameters), demonstrating that the device can be used feasibly and reliably in routine practice. This study of handheld SD OCT provided quantitative ONH parameter estimates with 95% prediction intervals for comparison of data from individual patients by a clinician using caliper measurements and also for future studies of pediatric ocular development and pathologic features (Tables 2 and 3).

### Normal Optic Nerve Head Development

**Figure 9:**
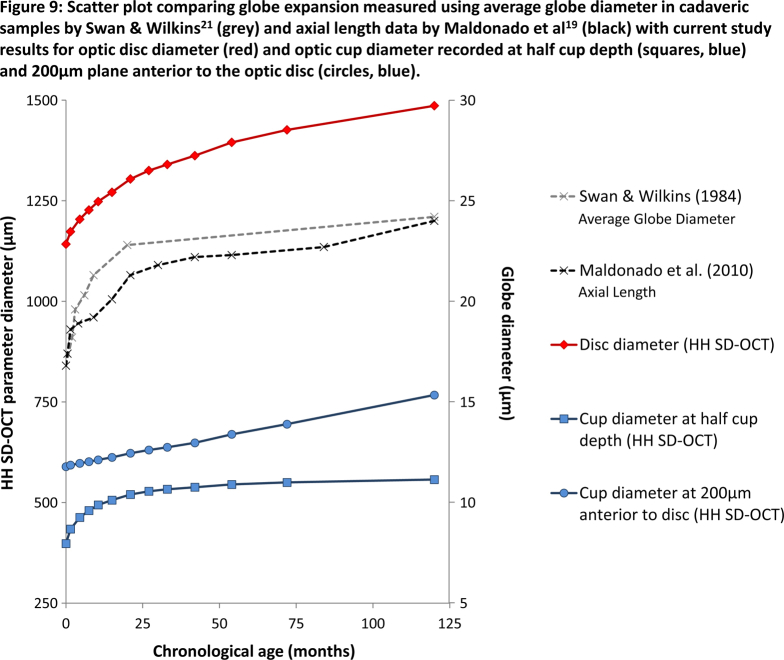


We report the size of the optic disc and cup diameter at term (postmenstrual age 40 weeks) is 76.9% and 71.4% of that observed in children aged 7-13 years (Fig 2A, C). Both cup and disc parameters demonstrated a nonlinear increase over time, particularly in the first few years of life. Figure 9 (available at www.aaojournal.org) compares changes of disc and cup diameters on a linear time scale with previously published findings of increasing globe diameter (average of sagittal, transverse, and vertical) from cadaveric data and in vivo biometric data of axial length.^19^, ^21^ Between birth and 2 years, disc and cup diameter demonstrated rapid increase with age-related changes in globe diameter. When expressed as a visual angle, we found no change in disc and cup diameters with age (Fig 2B, D), suggesting that parameters increase proportionally with increasing axial length. This agrees with previous observations made by Parsa^22^ that “as the globe expands in the first years of life, the scleral canal and thus the disk area can potentially expand slightly,” implying that expansion of the scleral shell is an important factor determining growth of the cup and disc in the first 2 years of life.

In a study of 95 cadaveric eyes, Rimmer et al^13^ describe a mean horizontal disc diameter of 1.13 mm for infants between 40 weeks' gestation and 6 months of age, increasing to 1.59 mm after 10 years of age. Our data demonstrated similar results of 1.14 mm at term and 1.49 mm between 7 and 13 years of age; however, direct comparison may be limited by the effect of formalin fixation and grouping of age ranges. More recently in a handheld SD OCT study of healthy full-term neonates, Allingham et al^23^ report average vertical disc and cup diameters of 1.29 to 1.38 mm and 0.44 to 0.56 mm, respectively. The results may be higher than reported in the current study (1.14 mm and 0.40 mm mean disc and cup diameters, respectively; Table 3) because of the effects of different scanning protocols, orientation, and analysis.

We found that the C/D_OCT_ remained stable between birth and 13 years of age within 0.381 to 0.386 using cup defined at half cup depth and within 0.483 to 0.498 using cup defined at 200 μm anterior to the disc plane (Fig 2E; Table 2). Previous literature reports differences in the size of cup-to-disc ratios in children measured using different imaging methods (i.e., fundus photography and table-mounted OCT), as well as conflicting results for changing optic cup-to-disc ratio with age.

Several funduscopy studies report small cup-to-disc ratios (C/D_fund_) at birth, typically with means of 0.1 or less, with few being more than 0.33, and cupless discs also being described.^22^, ^24^, ^25^ These values are much smaller than those defined using OCT, typically between 0.3 and 0.5 C/D_OCT_ from these data and previous studies highlighting the difficulty of directly comparing C/D_OCT_ with C/D_fund_.^15^, ^23^

In children younger than 2 years, only 1 study by Park et al^25^ describe longitudinal changes in C/D_fund_ measurements using serial ophthalmoscopy. This study of 73 full-term children describes a slow rate of C/D_fund_ progression of 0.0075 (95% confidence interval, 0.0049–0.0075) per year of age between birth and 10 years of age when described as a linear trend.^25^ In the current study using handheld SD OCT, we found no evidence of changing C/D_OCT_ in the first 4 years of life. However, we found that the optic cup was not measurable for 11 of 218 infants (5.5%) examined when the cup was measured at 200 μm anterior to the disc plane (see Fig 3Bii, available at www.aaojournal.org). All these infants were 4 years of age or younger. This suggests an increase in the percentage of measurable cups with age, and hence an increase in C/D_OCT_ in this subpopulation (i.e., increasing from 0 to a value of more than 0). To demonstrate this conclusively would require longitudinal studies.

Other studies have compared changes in C/D_OCT_ and C/D_fund_ in older children. McClelland et al^26^ found that children between 12 and 13 years of age had a larger C/D_fund_ than children 6 to 7 years of age when examined with fundus photography in a study of more than 400 participants. In contrast, Hellström et al^27^ found no difference in C/D_fund_ in 100 children between 2.9 and 19 years of age when also assessed with fundus photography. In older children able to comply with table-mounted OCT, the Sydney Childhood Eye Study compared ONH morphologic features in more than 2000 children 6 to 7 years of age and 12 to 14 years of age. Older children were found to have statistically larger optic disc diameters, but not optic cup diameters, with minimal change in C/D_OCT_ from 0.22 to 0.21.^28^ In a smaller study, Elia et al^10^ found no change in C/D_OCT_ when measured with Cirrus OCT (OCT3, Carl Zeiss Meditec, Dublin, CA) in 344 children 6 to 13 years of age.

### Development of the Peripapillary Region

No studies describe longitudinal changes in peripapillary RNFL in early years of life in children younger than 5 years, although several studies describe changes in children between 5 and 18 years of age in whom only a small degree of change in peripapillary RNFL is reported.^7^, ^8^, ^9^, ^10^, ^11^ Yanni et al^7^ and Turk et al^8^ found no significant change of RNFL with age for children between 6 and 16 years of age in studies including 83 and 107 participants, respectively. In a larger study of 470 children, Tsai et al^9^ found a small reduction in average RNFL thickness of 1.6% (*P* = 0.025) between 7 and 12 years of age. In contrast, we observed marked changes in the temporal RNFL in children younger than 5 years of age, with a reduction in thickness by 35% between birth and 12 months of age, followed by a 22% increase between 12 months and 13 years.

Video 1 (available at www.aaojournal.org) demonstrates how changes in RNFL thickness also need to be interpreted in the context of changes in the other optic parameters. The increasing size of the disc diameter seems to cause a lateral movement of the neuroretinal rim area and also centrifugal movement of the RNFL adjacent to the disc. Because there is a strong gradient in RNFL thickness as it approaches the disc (because the same number of fibers occupy an increasingly smaller volume), this centrifugal movement can create the impression of RNFL thickening. As with optic disc and cup widths, when RNFL is measured for a fixed visual angle, the changes with age follow a different dynamic, with only the temporal RNFL showing significant thinning in early development, but nasal RNFL remaining fairly constant (Fig 5B).

Tsai et al^9^ report that children 7 years of age had thicker RNFLs in all quadrants except temporally as compared with children 12 years of age. Similarly, Lee et al^29^ describe different correlation patterns of temporal RNFL with spherical equivalent, axial length, and age compared with global and all other quadrants for a wider age range (4–18 years). These studies suggest that temporal RNFL follows a different developmental trajectory than other regions. One reason for the apparent early temporal RNFL thinning could be that the rapid expansion of the scleral shell during this time causes distribution of nerve fibers over a wider surface area. However, this pattern is not repeated nasally, where the peripapillary RNFL seems to remain constant. One possibility is that the changing angle of insertion of the optic nerve into the scleral shell during orbital growth contributes to asymmetric changes in temporal and nasal peripapillary RNFL. Distortions of the disc and peripapillary RNFL resulting from oblique optic nerve insertion caused by myopic tilted discs were described several decades ago.^30^ More recently, Hwang et al^31^ quantified the asymmetric changes in temporal and nasal peripapillary RNFL associated with myopic tilted discs using OCT.

### Reliability

We report excellent interexaminer reproducibility of ONH parameters (Table 2), with ICC reported of more than 0.83 for diametric measures of all participants, including those younger than 4 years. These were comparable with the results from studies of older children imaged with table-mounted OCT systems.^11^, ^32^, ^33^ In a study of 100 children older than 6 years, Altemir et al^11^ report ICCs between 0.773 and 0.96 for RNFL, disc area, and rim area using Cirrus automated optic nerve analysis software. Smaller studies using a Stratus system report lower ICCs of between 0.63 and 0.85 for RNFL values, suggesting that sample size and use of different automated software may affect reliability of results.^32^, ^33^ As with earlier studies using either table-mounted or handheld OCT systems, we found lower reliability with RNFL measurements.^14^, ^32^, ^33^ This may be because of difficulty imaging the peripapillary region and the subsequent effect on image quality.

Comparison between right and left eyes demonstrated some variability of ONH morphologic features. Several studies of older children have reported physiologic variation of ONH parameters between eyes.^34^, ^35^ In the Sydney Childhood Eye Study including 1273 children 6 years of age, the C/D_OCT_ differed by less than 0.25 for 95% of participants. In contrast, the correlation for average RNFL was 0.72; however, it ranged between 0.34 and 0.55 for different quadrants, giving an intraocular difference of 47 μm in the quadrants.^34^ In a study of children 6 to 12 years of age, Altemir et al^35^ report similar results and suggest that C/D_OCT_ asymmetry of more than 0.25 between both eyes may be considered pathologic. We report varying symmetry between both eyes with an ICC of 0.74 for C/D_OCT_ and suggest that studies with larger patient groups adjusted for confounding factors including axial length be required to identify whether differences may be considered pathognomic.

Hwang et al^36^ describe the error rates generated using automatic detection algorithms of neuroretinal rim measurements where errors in temporal quadrants were found in 16 of 255 eyes (6.3%), mainly because of incorrect optic disc margin detection, and no detection errors were observed for nasal quadrants. In this study, we minimized detection errors using manual identification of the termination of Bruch's membrane and manual correction of automatic detection of ILM.

### Limitations

Potential limitations of the current study are confounding factors affecting data analysis. Although measurements were corrected for age^19^ and refractive errors of more than 3 D were excluded, the study did not calibrate for the true axial length and refractive error for each participant. Calibration of ocular OCT readings in children may be challenging because of increasing axial length and the ongoing process of emmetropization with age. Measurement of axial length often is difficult for young children unable to cooperate with fixation and centration. Refraction, a noncontact assessment that can be performed easily and quickly after dilation, often is used as an alternative. Axial length is accepted widely to be the most significant ocular determinant of refractive status; however, the importance may vary with age.^37^ Ip et al^38^ report that axial length accounts for 49% of variation in refractive error in 12-year-olds compared with only 24% in 6-year-olds, highlighting the dramatic changes in ocular development that occur with age. To our knowledge, there are no mathematical models to calibrate OCT readings accurately in young children for refraction, axial length, and age. To minimize any potential error caused by very high or low axial lengths, we excluded ±3.00 D, in keeping with normative databases for table-mounted OCT devices. In our dataset, spherical equivalent was not correlated to any of the ONH or peripapillary measured analyzed. Defining the lateral scales in terms of visual angle rather than as distance avoids the complexities of calibration caused by changes in axial length and refractive error during visual development.

Data analysis also is affected by limitations inherent with using OCT images to define ONH parameters. Reis et al^39^ describe different configurations of optic disc edge anatomic features with OCT, which may affect correct identification of Bruch's membrane particularly in infants in whom development is not yet complete.

The optic cup also may be defined using different reference planes (Fig 3A, B, available at www.aaojournal.org); however, no standardized methodology currently exists. Conventional table-mounted OCT devices, such as the RTVue-100 (Optovue, Inc, Fremont CA) and Cirrus (Carl Zeiss Meditec, Dublin, CA) often calculate optic cup diameter using a fixed reference plane relative to the disc plane (150 and 200 μm anterior to the disc plane, respectively). In this study, we defined the cup based on the excavation of the ONH (cup width at half cup depth), which we compared with the cup defined using a fixed reference plane relative to the disc plane. An excavation-based definition has the advantage of including all excavations of the ONH no matter how small, which shows less variability of C/D_OCT_ compared with a fixed reference plane relative to the disc plane (see Fig 3Aiv, Biv, available at www.aaojournal.org). An excavation-based definition of cup diameter also demonstrated a rapid increase in the first 2 years of life, in agreement with previous literature.

In this study, we did not compare either measures derived from funduscopy or from digital fundus photography with handheld SD OCT results. Samarawickrama et al^40^ compared digital planimetry results with table-mounted OCT results in 333 children 6.4 to 6.5 years of age and found that “OCT produced consistently smaller linear and area measures than digital planimetry,” also noting that “digital planimetry may be more sensitive than OCT in detecting small, physiologically normal optic cups and hence small cup/disc ratios.” However, Dai et al^41^ observed that 2-dimensional measurements of the optic disc with fundus imaging can lead to underestimates of horizontal and vertical disc measurements. These studies suggest that our results for ONH development may vary from previous published literature because of the potential differences between assessment and imaging methods.

Furthermore, the current study included analysis of a single B-scan through the deepest optic cup, preventing the superior and inferior quadrants of the optic nerve from being assessed. Further volumetric studies, including circumferential peripapillary RNFL and retinal thickness, are required to describe the optic nerve morphologic features in more detail.

In conclusion, we demonstrated the handheld SD OCT can be used to assess optic nerve morphologic features in infants and children without sedation and developed a pathway for use during routine clinical examinations. We showed high interexaminer reliability for measurement of ONH parameters via cross-sectional OCT analysis. Our findings correlate with established cadaveric, histologic, and fundus photography studies demonstrating that development continues throughout childhood. The most dramatic changes are seen within the first 2 years of life, when changes in global expansion are most apparent. Our normative database provides a comparison for future studies of children with ONH pathologic features.

## Figures and Tables

**Table 2:** Interexaminer Reproducibility and Intereye Variability of Optic Nerve Parameters Measured with Handheld Optical Coherence Tomography

Optic Nerve Parameter	Interexaminer Intraclass Correlation Coefficient	Intereye Intraclass Correlation Coefficient
Disc diameter	0.915 (0.947)	0.869 (0.892)
Cup diameter	0.892 (0.925)	0.793 (0.806)
Cup-to-disc ratio C/DOCT	0.830 (0.896)	0.741 (0.755)
BMO-MRW		
Nasal	0.980 (0.982)	0.872 (0.834)
Temporal	0.960 (0.979)	0.717 (0.712)
Retinal thickness		
Nasal	0.923 (0.957)	0.655 (0.636)
Temporal	0.907 (0.921)	0.793 (0.794)
RNFL		
Nasal	0.773 (0.657)	0.736 (0.505)
Temporal	0.681 (0.430)	0.713 (0.607)

BMO-MRW = Bruch's membrane opening–minimum rim width; RNFL = retinal nerve fiber layer.

Values in brackets are for participants younger than 4 years (n = 20). Of the 30 patients, 8 were younger than 1 year, 6 were 1–2 years of age, 6 were 2–4 years of age, and 10 were older than 4 years of age.

**Table 3:** Handheld Optical Coherence Tomography Values for Optic Disc Diameter, Cup Diameter, and Cup-to-Disc Ratio in Full-Term Children

Age	Axial Length (mm)	Disc Diameter (μm)	Cup Diameter (μm)	Cup-to-Disc Ratio
<1 wk	16.8	1142 (817–1468)	398 (116–680)	0.381 (0.212–0.549)
>1 wk–2.9 mos	18	1173 (848–1498)	434 (153–714)	0.381 (0.213–0.549)
3–5.9 mos	18.7	1204 (879–1529)	463 (183–743)	0.381 (0.213–0.549)
6–8.9 mos	19	1227 (902–1551)	480 (201–760)	0.381 (0.213–0.549)
9–11.9 mos	19.2	1248 (924–1573)	494 (214–773)	0.381 (0.213–0.549)
12–17.9 mos	20.1	1271 (947–1596)	506 (227–786)	0.381 (0.213–0.549)
18–23.9 mos	21.3	1304 (980–1629)	520 (241–800)	0.382 (0.214–0.55)
2–2.4 yrs	21.6	1325 (1001–1649)	528 (248–808)	0.382 (0.214–0.55)
2.5–2.9 yrs	21.8	1340 (1016–1665)	533 (253–812)	0.382 (0.214–0.55)
3–3.9 yrs	22.2	1362 (1037–1686)	538 (258–818)	0.382 (0.215–0.55)
4–4.9 yrs	22.3	1395 (1070–1720)	545 (265–825)	0.383 (0.215–0.551)
5–6.9 yrs	22.7	1426 (1100–1751)	550 (270–831)	0.384 (0.216–0.552)
7–13 yrs	24	1486 (1159–1812)	557 (277–838)	0.386 (0.216–0.556)

Data are mean values with upper and lower prediction intervals in parentheses.

**Table 4:** Handheld Optical Coherence Tomography Values for Optic Nerve Nasal and Temporal Retinal Nerve Fiber Layer and Nasal and Temporal Retinal Thickness in Full-Term Children

Age	Axial Length (mm)	Nasal Retinal Nerve Fiber Layer (μm)	Nasal Retinal Thickness (μm)	Temporal Retinal Nerve Fiber Layer (μm)	Temporal Retinal Thickness (μm)
<1 wk	16.8	50 (22.8, 77.2)	265.1 (230.4, 299.9)	70.5 (51.4, 89.6)	280.4 (246.4, 314.3)
>1 wk–2.9 mos	18	50.2 (23–77.4)	265.4 (230.7–300.1)	57 (38.2–75.8)	283.5 (249.6–317.3)
3–5.9 mos	18.7	50.3 (23.2–77.5)	265.7 (231–300.4)	49.3 (30.5–68.1)	286.4 (252.6–320.2)
6–8.9 mos	19	50.5 (23.4–77.7)	266 (231.3–300.7)	46.8 (28–65.6)	288.2 (254.5–322)
9–11.9 mos	19.2	50.7 (23.6–77.9)	266.3 (231.6–301)	45.6 (26.8–64.5)	289.9 (256.2–323.7)
12–17.9 mos	20.1	50.9 (23.8–78.1)	266.7 (232.1–301.4)	45.5 (26.7–64.3)	291.6 (257.8–325.3)
18–23.9 mos	21.3	51.4 (24.2–78.5)	267.5 (232.9–302.2)	46.3 (27.5–65)	293.6 (259.8–327.3)
2–2.4 yrs	21.6	51.7 (24.6–78.8)	268.1 (233.4–302.7)	47.1 (28.4–65.9)	294.7 (261–328.5)
2.5–2.9 yrs	21.8	51.9 (24.8–79)	268.5 (233.9–303.2)	47.9 (29.1–66.6)	295.5 (261.8–329.3)
3–3.9 yrs	22.2	52.4 (25.3–79.5)	269.3 (234.7–304)	49 (30.2–67.7)	296.6 (262.8–330.3)
4–4.9 yrs	22.3	53.2 (26.1–80.3)	270.8 (236.2–305.5)	50.8 (32.1–69.6)	298 (264.3–331.8)
5–6.9 yrs	22.7	54.3 (27.1–81.4)	272.7 (238–307.4)	52.5 (33.7–71.3)	299.2 (265.4–333.1)
7–13 yrs	24	57.2 (29.7–84.7)	277.8 (242.6–312.9)	55.5 (36.6–74.4)	301.2 (267.4–335.1)

Data are mean values with upper and lower prediction intervals in parentheses.

## Supplementary Data

Table 1

Video 1 Video Clip of schematic video showing optic nerve head development from birth to adolescence.
