# Supplementary material for: Optic Nerve Head Development in Healthy Infants and Children Using Handheld Spectral-Domain Optical Coherence Tomography
Source: Ophthalmology. 2016 Oct;123(10):2147–57. doi: 10.1016/j.ophtha.2016.06.057 (PMC5036922; doi:10.1016/j.ophtha.2016.06.057)
Supplement: Table 1 [file mmc1.pdf]

Table 1: Percentage of successful handheld spectral domain optical coherence tomography (HH SD-OCT) optic nerve images categorized by age group

Age	Number of participants	Successful optic nerve scan, n (%)
**<1 week**	30	20 (67)
**>1 week – 2.9 months**	34	21 (62)
**3 – 5.9 months**	33	27 (81)
**6 – 8.9 months**	32	23 (72)
**9 – 11.9 months**	25	19 (76)
**12 – 17.9 months**	32	17 (53)
**18 – 23.9 months**	26	14 (54)
**2 – 2.49 years**	20	12 (60)
**2.5 – 2.99 years**	15	10 (67)
**3 – 3.99 years**	23	16 (70)
**4 – 4.99 years**	22	15 (69)
**5 – 6.99 years**	39	34 (87)
**7 – 13 years**	21	21 (100)
