# Supplementary figures and images for: Optic Nerve Head Development in Healthy Infants and Children Using Handheld Spectral-Domain Optical Coherence Tomography
Source: Ophthalmology. 2016 Oct;123(10):2147–57. doi: 10.1016/j.ophtha.2016.06.057 (PMC5036922; doi:10.1016/j.ophtha.2016.06.057)

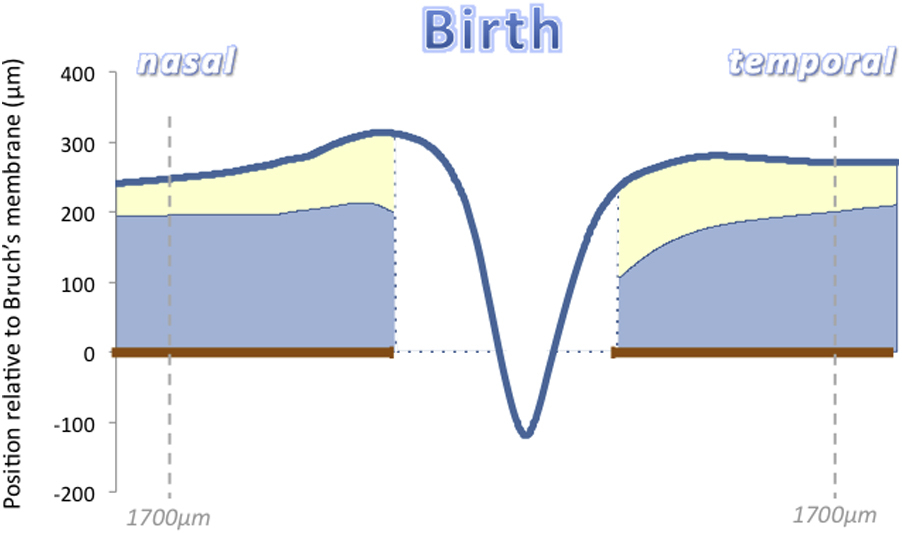

Supplement: Video 1 — Video Clip of schematic video showing optic nerve head development from birth to adolescence. [file mmc2.jpg]
